# Emotional Stress State Detection Using Genetic Algorithm-Based Feature Selection on EEG Signals

**DOI:** 10.3390/ijerph15112461

**Published:** 2018-11-05

**Authors:** Dongkoo Shon, Kichang Im, Jeong-Ho Park, Dong-Sun Lim, Byungtae Jang, Jong-Myon Kim

**Affiliations:** 1 School of Computer Engineering and Information Technology, University of Ulsan, Ulsan 44610, Korea; dongkoo88@gmail.com; 2 ICT Convergence Safety Research Center, University of Ulsan, Ulsan 44610, Korea; kichang@ulsan.ac.kr; 3 Industry IT Convergence Research Group, Intelligent Robotics Research Division, SW Contents Research Laboratory, Electronics and Telecommunications Research Institute (ETRI), Daejeon 34129, Korea; parkjh@etri.re.kr (J.-H.P.); dslim@etri.re.kr (D.-S.L.); jbt@etri.re.kr (B.J.)

**Keywords:** stress detection, *k*-nearest neighbors, genetic algorithm, machine learning

## Abstract

In recent years, stress analysis by using electro-encephalography (EEG) signals incorporating machine learning techniques has emerged as an important area of research. EEG signals are one of the most important means of indirectly measuring the state of the brain. The existing stress algorithms lack efficient feature selection techniques to improve the performance of a subsequent classifier. In this paper, genetic algorithm (GA)-based feature selection and *k*-nearest neighbor (*k*-NN) classifier are used to identify stress in human beings by analyzing electro-encephalography (EEG) signals. GA is incorporated in the stress analysis pipeline to effectively select subset of features that are suitable to enhance the performance of the *k*-NN classifier. The performance of the proposed method is evaluated using the Database for Emotion Analysis using Physiological Signals (DEAP), which is a public EEG dataset. A feature set is extracted in 32 EEG channels, which consists of statistical features, Hjorth parameters, band power, and frontal alpha asymmetry. The selected features through GA are used as input to the *k*-NN classifier to distinguish whether each EEG datapoint represents a stress state. To further consolidate, the effectiveness of the proposed method is compared with that of a state-of-the-art principle component analysis (PCA) method. Experimental results show that the proposed GA-based method outperforms PCA, with GA demonstrating 71.76% classification accuracy compared with 65.3% for PCA. Thus, it can be concluded that the proposed method can be effectively used for stress analysis with high classification accuracy.

## 1. Introduction

Since many industrial accidents occur due to the unsafe behaviors of workers, adequate precautions are needed to analyze in depth the causes of human error. Factors leading to unsafe behavior include fatigue, sleep deprivation, stress, and physical defects. Stress is defined as the body’s response to mental, emotional, or physical pain. Stressful conditions not only cause unstable behaviors but can also aggravate hypertension or coronary artery disease if the stress persists [1]. According to [2], stress is also related to diseases such as irritable bowel syndrome and depression.

Stress also affects the body’s respiration and circulation. Stressful conditions increase both breathing and heart rates. These emotional changes can also change brain activity, which can be detected by functional magnetic resonance imaging (fMRI), near-infrared spectroscopy (NIRS), electrocorticography (ECoG), and electroencephalography (EEG).

Various methods of obtaining signals from the brain are introduced in the literature [3]. fMRI uses magnetic fields, and NIRS uses near-infrared to measure brain activation using brain blood. fMRI has the advantage of measuring signals inside the brain and has an excellent spatial resolution, but the measurements are delayed until the state of the brain changes, requiring relatively large equipment. NIRS measures brain activity at a lower cost relative to fMRI, but it has a disadvantage that it can only detect the state of the brain surface and its signal is indirectly obtained through blood flow. ECoG and EEG are used to measure brain waves. ECoG is a method of obtaining brain waves by attaching electrode pads directly to the brain surface. It has the advantage of achieving high resolution and wide bandwidth. However, electrode placement on the surface of the brain requires surgery. On the other hand, EEG measures brain waves via electrodes placed on the scalp hence surgery. However, EEG can measure only the activity of the cortex region in the brain, and it is vulnerable to noise. According to [4], EEG is non-invasive and has the advantages of relatively easy signal acquisition and high time resolution. Since EEG acquires signals from the scalp, it is easy to integrate it as a wearable technology through coupling with a helmet, which can be used to protect the wearer in the case of industrial accidents. Bio-signals and EEG measurement approches via helmet were proposed in a study in [5].

Wheeler et al. [6] observed a negative emotional state when the right hemisphere activity of the cerebral cortex is active during the stress period, and several studies showed that researchers investigate a correlation between EEG measurements and depression. Based on these studies, Atencio et al. [7] used the frontal alpha asymmetry index to obtain the emotional stress state using the EEG public database and classified them using feature extraction and SVM. Bastos-Filho et al. [8] extracted signal features to detect stress based on EEG signals and classified them using *k*-NN. However, in [7,8], a large number of features were used to classify, which generally causes a problem called the curse of dimensionality. With many features, the size of the learning set required for modeling must be proportionally large, which takes a long time to categorize. Accordingly, various manifold learning approaches such as PCA (principal components analysis), maximal revision minimum redundancy (mRMR) [9], and various feature space reduction techniques such as selecting features using F-scores have been used to address the issue [10]. The existing stress analysis methods lack in an effective features dimensionality reduction technique to improve performance of a subsequent classifier. In this paper, GA-based feature selection for reduction of the EEG feature space is introduced. In addition, the classification performance of the proposed method is compared with PCA. GA ensures that the selected features subset is well suited to enhance the classification performance. GA can easily decide to select or not a feature in the subset as sequences of Boolean operations. It allows the algorithm to efficiently explore the original feature space by retraining just those instances that boost the classification performance. Moreover, it also avoids the local optima caused by the intrinsic randomness of the original feature space. The experimental results of the proposed method provide 71.76% of classification accuracy which is 6.46% higher than that of the PCA based stress analysis method.

The remainder of this paper is organized as follows: Section 2 describes the entire process, dataset, and algorithms for stress classification. GA-based feature selection is used to classify stress and non-stress states, and it is compared with PCA by classifying them in the *k*-Nearest Neighborhood (*k*-NN). Section 3 validates the performance of feature selection algorithms such as PCA and GA-based feature selection. Finally, Section 4 concludes this paper.

## 2. Materials and Methods

### 2.1. An Overall Process of Stress Classification

**Figure 1:**
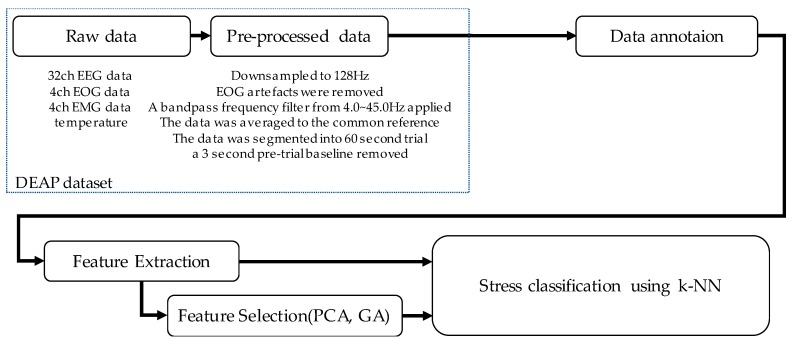
An overall process of stress classification.

The overall process for stress classification is shown in Figure 1. To classify stress state, the following four steps are used: data annotation, feature extraction, feature selection, and classification. Data annotation labels the stress state and calm state. Feature extraction calculates features that can well classify states in a classification algorithm. Feature selection reduces the dimensions of the feature space, and stress classification classifies stress and calm state using the selected features.

### 2.2. Dataset

Database for Emotion Analysis using Physiological Signals (DEAP) [11], a public EEG data set was used in this paper. Thirty-two healthy participants were shown 40 different music videos each 1-min long for emotional stimulation and acquired EEG when watching music videos. In summary, in DEAP emotional state was collected from arousal and valence through self-assessment manikins (SAM) [12]. The dataset contains pre-processed data that downsamples the original data to 128 Hz, applying a 4 Hz to 45 Hz bandpass filter and removing EEG artifacts. In this paper, we have used pre-processed data provided by DEAP.

### 2.3. Data Annotation

For the experiment, the stress and the calm states are defined according to the rule used in [8]. If arousal is less than 4 and valence is between 4 and 6, as in Equation (1), it is defined as calm:

(1) (arousal<4)∩(4<valence<6) 

where *arousal* stands for a range from calm to excited, while *valence* presents a range from unpleasant to pleasant.

If arousal exceeds 5 and valence is less than 3, as in Equation (2), it is defined as a stress state:

(2) (arousal>5)∩(valence<3) 

**Figure 2:**
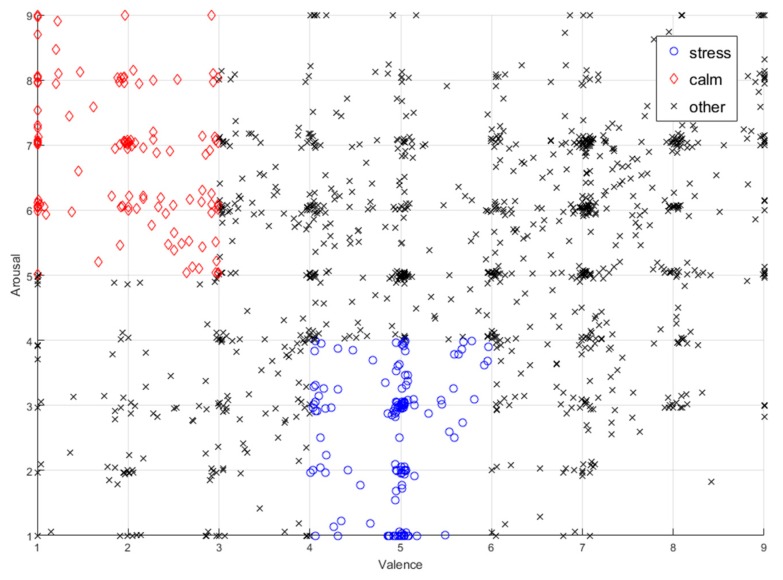
The result of data annotation.

After separating the stress and calm states for each participant, seven out of the 32 participants did not exhibit both a distinctive stress state and calm state, there these participants could not be used as learning data for classification. Therefore, an experiment is conducted for the remaining 25 participants. Figure 2 shows the result of the data annotation process.

### 2.4. Feature Extraction

Pre-processed EEG signals are separated according to each trial, and the EEG signal length of each trial is about 60 s (8064 samples). Since there are only 40 trials per participant, there is not enough learning data for a machine learning algorithm. Therefore, the 8064 samples are divided into 16 parts, and data segments of about 4 seconds in length (504 samples) are used for the experiment.

The features extracted are statistical features, frequency domain features, higher-order crossings, Hjorth parameter, and frontal asymmetry alpha, all of which are widely used in EEG analysis in related research [8,10,13,14,15,16,17,18,19].

#### 2.4.1. Statistical Features

In this paper, the following six features described in [8,13] are used as statistical features:

- Mean: $\mu_{x}=\frac{1}{N}\sum_{n=1}^{N}X(n)$
- Standard deviation: $\sigma_{x}=\sqrt{\frac{1}{N}\sum_{n=1}^{N}{\left(X(n)-\mu_{x}\right)}^{2}}$
- First difference: $\delta_{x}=\frac{1}{N-1}\sum_{n=1}^{N-1}\left|X(n+1)-X(n)\right|$
- Normalized first difference: ${\tilde{\delta }}_{x}=\frac{\delta_{x}}{\sigma_{x}}$
- Second difference: $\gamma_{x}=\frac{1}{N-2}\sum_{n=1}^{N-2}\left|X(n+2)-X(n)\right|$
- Normalized second difference: ${\tilde{\gamma }}_{x}=\frac{\gamma_{x}}{\sigma_{x}}$

Here, *X*(*n*) is data obtained by dividing the pre-processed EEG signal of the DEAP data set by 16, and thus *N* = 504. Since six features are extracted for each of 32 channels of EEG, the number of statistical features extracted per experiment is 192.

#### 2.4.2. Frequency Domain Features

The most commonly used feature of emotion recognition research using EEG is the power per frequency band. Generally, EEG is divided into five bands, designated as delta, theta, alpha, beta, and gamma [10,14,15]. Depending on the researcher’s definition, the range of each frequency domain can be adjusted slightly. According to Sanei et al. [20], delta waves are associated with deep sleep, and theta waves are mainly produced in the sleepy state. The alpha wave reflects the state of being awake comfortably, and the beta wave is mainly associated with the concentrated state. Gamma waves appear rarely. In this paper, seven bands are used for the experiment in [8]. Table 1 lists the seven bands used. Band power for these seven bands is calculated as the frequency domain features. Band power is obtained by calculating the power spectrum density (PSD) using Welch’s method [21] and then summing the frequency band ranges. Welch’s method divides the time domain data into overlaid sections and then averages the spectra for each section, thereby reducing the effects of temporarily unstable signals or noise. The PSD feature was used in [8,14].

Since seven features are extracted for each of 32 channels of EEG, the number of frequency domain features extracted per experiment is 224.

#### 2.4.3. Higher-Order Crossings (HOC)

According to [16], higher-order crossing (HOC) is related to the emotional state. HOC is useful for indicating the pattern of periodic change of the EEG signal.

Zero-crossing is the count of zero-crossing signals, and HOC counts the various high-pass filtered time series using the difference operator. Since the calculation is simple, spectrum analysis can be performed efficiently.

The HOC features are defined by the sequence of *D* as follows:

(3) $$\mathrm{HOC}=[D_{1},D_{2},\ldots ,D_{M}],$$

where *M* is the maximum value of the HOC order in the feature vector; *M* = 5 is used in this paper.

#### 2.4.4. Hjorth Parameter

The Hjorth parameter [17] is a feature consisting of activity, mobility, and complexity that can be obtained in the time domain. Activity is the variance of the signal amplitude and represents the average power. Mobility is the square root of the variance of the signal magnitude in the variance of the signal slope and represents the mean frequency. Complexity indicates how closely the signal resembles a pure sine wave.

This parameter is calculated in the time domain but contains information about the frequency spectrum of the signal. Therefore, it has a lower computation time than other methods of obtaining information on the frequency spectrum.

The Equation for obtaining each parameter in the discrete signal is as follows:

(4) $$\sigma_{1}=\frac{1}{n}\sum_{i=1}^{n}x_{i}^{2},$$

where *x* denotes signal, and *σ*_1_ stands for variance of *x*.

(5) $$\sigma_{2}=\frac{1}{n-1}\sum_{i=1}^{n-1}{\left(x_{i+1}-x_{i}\right)}^{2},$$

where *σ*_2_ stands for derivative of variance.

(6) $$\sigma_{3}=\frac{1}{n-2}\sum_{i=1}^{n-2}{\left((x_{i+2}-x_{i+1})-(x_{i+1}-x_{i})\right)}^{2},$$

where *σ*_3_ is 2nd order derivative of variance.

(7) $$activity(f(i))=\mathrm{var}iance(f(i))=\sigma_{1},$$

where *activity* is a measure of the variance of a signal.

(8) $$mobility(f(i))=\sqrt{\frac{activity\left(\frac{df}{di}\right)}{activity(f(i))}}=\sqrt{\frac{\sigma_{2}}{\sigma_{1}}},$$

where *mobility* denotes the signal’s mean frequency.

(9) $$complexity(f(i))=\frac{mobility\left(\frac{df}{di}\right)}{mobility(f(i))}=\sqrt{\frac{\sigma_{3}/\sigma_{2}}{\sigma_{2}/\sigma_{1}}}.$$

where *complexity* stands for deviation of the signal from the sine shape.

#### 2.4.5. Frontal Asymmetry Alpha

The results of several studies [18,19,22,23,24,25] revealed abnormal brain waves in patients with depression. Especially in the cerebral hemisphere, the frontal lobes of the right hemisphere are asymmetrical in the left hemisphere rather than the right hemisphere.

Fp1 and Fp2 are used in EEG electrodes to see the difference between the frontal lobes. Fp refers to the pre-frontal region in the brain. The power of the alpha wave band is obtained from each electrode, and the absolute value and the log are taken and subtracted from each other.

The formula to obtain frontal asymmetry alpha is as follows:

(10) Frontal Asymmetry Alpha=|lnR|−|lnL|.

where *R* and *L* stand for signal power for right and left hemisphere, respectively.

#### 2.4.6. Features Set for Classification

In the DEAP data set, the EEG signals are 32 channels, and the features are extracted for each channel. In the case of frontal asymmetry alpha, however, there is only one feature because it uses only *Fp*1 and *Fp*2 positions. Table 2 shows the number of features extracted for each feature type.

### 2.5. Feature Selection

Feature selection is a way to reduce a high-dimensional feature space. It is possible to reduce the computation time for the classification through the feature selection. In this paper, principal component analysis (PCA) and GA-based feature selection are compared.

PCA [26] is commonly used in feature dimensionality reduction. This method is useful for visualizing a high-dimensional feature space that is interrelated by finding a principal component (PC) that maximizes the variance of the features. In this way, redundant information from the original features space is eliminated. In PCA, a transformation matrix is used to scale and rotate the original features space. It can be formulated as a linear transformation by projecting feature vectors on transformed subspace by relevant directions. PCA generalizes the feature selection process for the whole set of input data.

Genetic algorithms (GAs) [27] are a robust and efficient optimization technique based on natural evolutionary theory. GA consists of major operations such as selection, crossover, mutation, and replacement. A GA-based feature selection selects the best combination of the most distinguishable classes as a good chromosome.

It is necessary to calculate the degree of separation between the two classes in order to find the feature combination that distinguishes between the classes. An equation for the degree of separation between classes is shown in Equation (11):

(11) $$\mathrm{class}\_\mathrm{separability}\_\mathrm{rating}=\frac{\mathrm{inter}\_\mathrm{classes}\_\mathrm{separability}}{\mathrm{intra}\_\mathrm{class}\_\mathrm{closeness}},$$

where inter_classes_separability is a measure of how far apart different classes are, and the intra_class_closeness is a measure of how close the features within the same class are. As a result, a high class_separability_rating is achieved when inter_classes_separability is maximized and/or when intra_class_closeness is minimized.

In this study, the Euclidean distance is used to find the distance between two feature vectors. Equation (12) shows the Euclidean distance formula:

(12) $$d(x,y)=\sqrt{{\sum_{i=1}^{n}\left(x_{i}-y_{i}\right)}^{2}},$$

where *x* and *y* are two different feature vectors, and n is a number of features.

The inter_classes_separability calculates average distance of each feature vector of different classes. The distances between all the feature vectors of two different classes are calculated and then averaged. The Equation is shown as follows:

(13) $$\mathrm{inter}\_\mathrm{classes}\_\mathrm{separability}=\frac{1}{{}_{N}c_{2}\cdot M\cdot M}\sum_{i=1}^{N}\sum_{j=i+1}^{N}\sum_{k=1}^{M}\sum_{l=1}^{M}d(i_{k},j_{l}),$$

where intra_class_closeness is a value obtained by calculating all the distances between feature vectors among the features of the same class and then averaging them. Equation (14) shows how to calculate intra_class_closeness:

(14) $$\mathrm{intra}\_\mathrm{class}\_\mathrm{closeness}=\frac{1}{N\cdot M\cdot M}\sum_{i=1}^{N}\sum_{j=1}^{M}\sum_{k=1}^{M}d(i_{j},i_{k}),$$

In Equations (13) and (14), *N* is the numbers of classes. In this paper, there are two classes (stress and calm), and thus, *N* = 2. The number of feature vectors for each class are denoted as *M*. In both Equations *d*(*x*,*y*) represents the distance among the difference classes or among different features with in the class, and *i_j_* means *j*-th feature vector in *i*-th class.

### 2.6. Classification

In this paper, *k*-nearest neighbors (*k*-NN) [15] is used for classification. The *k*-NN classifier is one of the most popular classification schemes due to its simplicity and computational efficiency. It classifies the corresponding classes by comparing the features from the feature extraction and feature selection process with the closest *k* learning data. To reduce the possibility of specific results for specific learning data, we use *k*-fold validation to separate training and test data (i.e., *k* = 3 in this study).

## 3. Experimental Results and Discussion

Table 3 shows classification accuracies of three different experiments for stress analysis, i.e., classification by means of *k*-NN classifier without any feature selection, features selection through PCA, and features selection through GA. The three experiments are repeated multiple times, and the best-case accuracies for all the experiments are included in the table. The GA-based feature selection algorithm shows the highest performance in most of the cases, i.e., for 17 participants (68% of 25 subjects). Whereas, the classification accuracy is higher for seven participants when all the extracted features are used for the classification purpose without embedding any feature section scheme into the stress analysis pipeline. PCA yields satisfactory results with higher accuracy than the other two arrangements just in the case of one participant. Moreover, there is at most 1.18% difference in the accuracies for the case where GA-based feature selection exhibits relatively lower performance. Overall, the results suggest that performance of the GA-based feature selection algorithm is satisfactory. The reason behind the relatively better performance is that GA just selects those features in the reduced features set that are good for the classification task.

**Figure 3:**
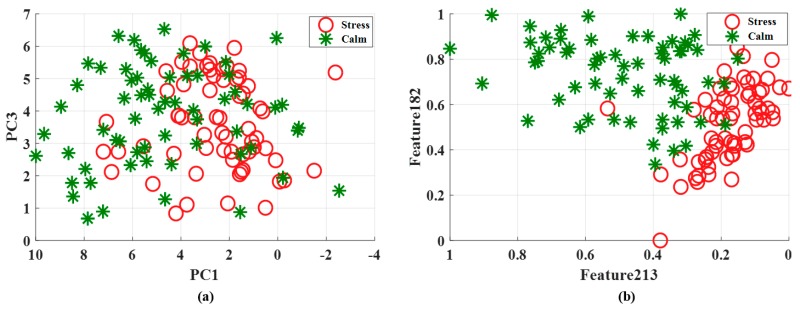
Comparison between (**a**) PCA and (**b**) GA-based feature selection.

Figure 3 shows the scatter plot of features selected through PCA and GA for a single participant (i.e., participant number 15) where the difference of the classification accuracies between the two algorithms is significant. It is evident from the figure that features selected through PCA for two classes are undistinguishable and fail to create distinct clusters. On the other hand, the results of the GA-based feature selection can visually confirm that the separation between the classes is relatively good.

Table 4 shows a comparison of the performance of feature selection algorithms. The average accuracy is 67.08% for all features, 65.03% for features selected using PCA, and 71.76% for features using GA-based features. Experimental results show that the best classification performance is obtained when using GA-based feature selection. The results suggest that inter class features representation is better when GA-based feature selection is used than when all features are used, therefore, enhancing the performance of the *k*-NN classifier.

The results suggest that the overall performance of the stress analysis pipeline is improved when a suitable feature selection technique is introduced in the pipeline. It not only improves the classification performance of a subsequent classifier but also reduces the input feature space avoiding the computation overhead caused by redundant information presented in the input feature space. Although incorporation of the GA-based feature selection technique improves the performance of the stress analysis pipeline, it has a room for improvement. The EEG signals contain different types of artifacts that make the feature extraction for stress analysis a challenging task. An artifact is a disturbance in the measured brain signal. The artifact signal which is not originated from the brain comes from other internal or external sources. The performance can be further improved if suitable preprocessing technique is integrated in the stress analysis pipeline, which can separate the unwanted information from the brain signals. The features extracted from the artifact free EEG signals associated with different class can make more vibrant clusters. As a result, it can further improve the performance of a subsequent classifier.

## 4. Conclusions

In this paper, a stress analysis technique was introduced to classify whether the EEG signal represents a stress state or calm state. Several types of features that can define the inter class representation were extracted from different domains. Then, a reduced features set was acquired from the original features space by using the GA and PCA based feature selection methods. The classification was carried out with the virtue of the *k*-NN algorithm by using the reduced feature subsets through PCA and GA. Finally, the classification accuracy was measured. Experimental results showed that classification performance is best when the proposed GA-based method was used. When all features were used, the accuracy was 67.08% with the *k*-NN classifier. The accuracy of feature space reduction using PCA with the *k*-NN classifier was 65.03%, while the accuracy of GA-based feature selection with the *k*-NN classifier showed 71.76%. This suggests that the proposed GA-based method provides well-chosen features that distinguish between classes.

## Figures and Tables

**Table 1:** Frequency band ranges of recorded EEG signals.

No. of Frequency Band	Frequency Range (Hz)
1	4–7.2 Hz
2	7.2–10.4 Hz
3	10.4–13.8 Hz
4	13.8–17 Hz
5	17–20 Hz
6	20–23 Hz
7	23–60 Hz

**Table 2:** Number of features from each type using the EEG signals.

Feature Types	Number of Features
Statistical features	192
Frequency domain (Power spectral density)	224
HOC	160
Hjorth Parameter	96
Frontal Asymmetry Alpha	1

**Table 3:** Classification performance of the three different methods for feature selection.

Participant No.	All Features + KNN Precision	PCA + KNN Precision	GA Based + KNN Precision
1	87.91%	80.10%	87.13%
2	63.91%	60.22%	61.64%
4	54.69%	51.04%	52.60%
5	61.24%	59.36%	68.15%
8	72.54%	66.93%	72.51%
10	60.05%	55.14%	61.83%
11	68.03%	66.59%	75.72%
12	73.58%	73.29%	72.45%
13	71.63%	72.83%	78.84%
14	82.21%	80.29%	83.65%
15	79.31%	75.80%	91.42%
16	62.24%	60.16%	64.84%
18	73.48%	73.44%	79.68%
19	51.84%	49.98%	61.22%
20	58.98%	54.28%	62.49%
21	66.78%	64.45%	72.64%
22	63.97%	62.14%	62.14%
24	73.26%	75.00%	77.08%
25	60.94%	61.14%	60.56%
26	54.69%	54.62%	89.11%
27	88.89%	88.54%	88.19%
28	63.54%	65.63%	68.23%
29	67.19%	64.06%	68.23%
31	54.69%	51.82%	57.81%
32	61.35%	58.99%	75.78%

**Table 4:** Comparison of average precision of related stress classification from the EEG recording.

Features	Classifier	Precision
all features	*k*-NN	67.08%
PCA [26]		65.03%
proposed GA method		71.76%
